# Commencement in Rush Medical College

**Published:** 1864-02

**Authors:** 


*Commencement in Rush Medical College.*—The commencement exercises of the twenty-first annual course of lectures in Rush Medical College were held on Wednesday evening, the 27th ult., on which occasion seventy-nine gentlemen received the degree of Doctor of Medicine. Prof. Brainard, President of the Faculty, conferred the degrees, presenting each graduate with his diploma—after which he delivered a most instructive and pleasing valedictory address. We hope to have the pleasure of presenting it to the readers of the *Journal*, feeling sure its perusal would gratify the friends of the author, and of the College.

The course of lectures thus brought to so auspicious a close, has been more gratifying than any since the organization of the institution. In literary attainments and gentlemanly deportment, as well as in numbers, the class has been one of which the Faculty of any medical college might well be proud. The prospects of the College are brighter than its most sanguine friends, at the re-organization of the Faculty five years since, had dared to hope. And they would have been considered visionary if they had predicted that the twenty-first course would be attended by more than two hundred and fifty students, but this is the fact. As proof of the completeness of the course, and thoroughness of teaching, we may refer to the uniform success, and honorable position of the alumni, who may be found occupying responsible places in every section of the country and in every division of the army. We subjoin the list of the graduating class:

| NAMES. | SUBJECT OF THESIS. |
|---|---|
| Adkins Frank B.... | The Human Skeleton. |
| Akely Harrison.... | Treatment of Typhoid Fever. |
| Allen Orlenzer.... | Digestion. |
| Avery Samuel J.... | Version. |
| Babcock Lyman F.... | Æration of the Blood. |
| Babcock Charles M.... | Colic. |
| Bradbury William T.... | Cholera Infantum. |
| Bibb George R.... | Burns and Scalds. |
| Byrn Spencer.... | Typhoid Fever. |
| Bacon A. J.... | Diphtheria. |
| Beasley G. Frank.... | Blood. |
| Bucher Charles A.... | Chronic Diarrhœa. |
| Cass Frank D.... | Remittent Fever. |
| Coakley James E.... | Hœmorrhage. |
| Chamberlain Ellston.... | ——— |
| Dayton Ephraim.... | Syphilis. |
| Dora James W.... | Congestive Continued Fever. |
| Dora F. Beauchamp.... | Malaria. |
| Eells Franklin.... | Pneumonia. |
| Egbert J. Wesley.... | Malarious Disease. |
| English F. Edwin.... | Pre-requisites to the Practice of Medicine. |
| Fares J. B.... | Diphtheria. |
| Gaylord Horace.... | Circulation of the Blood. |
| Glasener E. T.... | Inguinal Hernia. |
| Goodwin Lewis H.... | Dysentery. |
| Goldsbury J. A.... | Diphtheria. |
| Hiatt J. Milton.... | Gonorrhœa. |
| Hill Robert Lewis.... | Gun Shot Wounds. |
| Hallingsworth H. C..... | Inguinal Hernia. |
| Jordon Frank A..... | Diphtheria. |
| Jones Erwin L..... | Ovum and its Development. |
| Jones Augustus P. C..... | Colo Rectitis. |
| Johnson I. C..... | Dysentery. |
| Jennings George N..... | Vis Medicatrix Natura. |
| Kelly John J..... | Reflex Action of the Spinal Cord. |
| Keely Leslie E..... | Management of Labor. |
| Kelso Robert S..... | The Atmosphere. |

Kerrell John R.....Obstetrics.
Kinnear A. H.....Incised Wounds.
Larimer Bartlett....Typhoid Fever.
Lester Gilbert B.....Diphtheria.
Linn Timothy T.....Management of the Sick Room.
Lowell Lorenzo Dow....Scarlatina.
Lyons J. Elles....Contagion.
Macdonald Peter S.....Intermittent Fever.
McGlumphy S. B ....Evidences of Gestation.
Marion Francis....Alcohol.
Mendenhall Samuel....The Incentives and Duties of the Medical Profession.
Mix Henry A.....Opium.
Munger Martin E.....Stone in the Bladder.
Munroe James A.....Signs of Pregnancy.
Moses Jabez H.....The Liver.
Nelson Alexander P.....Placenta Prævia.
Nelson Eugene L.....Medical Science, its Progress and Lessons.
O'Brien J. N.....Diarrhœa.
Palmer Roswell R.....Bilious Remittent Fever.
Peebles G. Hial....Menstruation.
Price Edward H.....Cause and Effect in their relation to Medical Science.
Richardson Charles M.....Circulation of the Blood.
Shaffer Philip....Typhoid Fever.
Schuchard George W.....Pernicious Fever.
Smith William A.....Ununited Fractures.
Still J. M.....Diphtheria.
Stillman J. Dwight....Physical and Chemical Action in Science.
Swift John M.....——
Thayer John W.....The Accoucheur.
Tevis Joel T.....Malarial Fever.
Waterhouse Marvin....Diphtheria.
West John M.....Malaria.
Welsh William F.....Diphtheria.
Wilson Samuel....Pneumonia.
Williams J. A.....Pathology of Fever.
White Charles A.....Classification of Medicine.
Watkins James M.....Intermittent Fever.
Wood Orlando S.....Anatomy and Physiology of the Liver.
Yerkes Titus P.....Signs of Pregnancy.

The *ad eundum* degree of M. D. was conferred on Charles White, M. D., and Frederick S. C. Grayston, M. D.

